# Scrutinizing different predictive modeling validation methodologies and data-partitioning strategies: new insights using groundwater modeling case study

**DOI:** 10.1007/s10661-024-12794-w

**Published:** 2024-06-17

**Authors:** Alvin Lal, Ashneel Sharan, Krishneel Sharma, Arishma Ram, Dilip Kumar Roy, Bithin Datta

**Affiliations:** 1 Global Centre for Environmental Remediation, College of Engineering, Science and Environment, The University of Newcastle, Callaghan, New South Wales Australia; 2 CRC for Contamination Assessment and Remediation of the Environment (crcCARE), The University of Newcastle, Callaghan, New South Wales 2308 Australia; 3 Dicipline of Civil Engineering, College of Science & Engineering, James Cook University, Townsville, Australia; 4 C&R Consulting, Geochemical and Hydrobiological Solutions Pty Ltd, Aitkenvale, Queensland 4814 Australia; 5 School of Environmental and Life Sciences, College of Engineering, Science and Environment, The University of Newcastle, Callaghan, New South Wales Australia; 6 School of Agriculture, Geography, Environment, Ocean and Natural Sciences, University of the South Pacific, Laucala Campus, Suva, Fiji; 7 Irrigation and Water Management Division, Bangladesh Agricultural Research Institute, Joydebpur, Gazipur, 1701 Bangladesh

**Keywords:** Groundwater salinity, Machine learning, GMDH, FEMWATER, Data partitioning strategies

## Abstract

Groundwater salinity is a critical factor affecting water quality and ecosystem health, with implications for various sectors including agriculture, industry, and public health. Hence, the reliability and accuracy of groundwater salinity predictive models are paramount for effective decision-making in managing groundwater resources. This pioneering study presents the validation of a predictive model aimed at forecasting groundwater salinity levels using three different validation methods and various data partitioning strategies. This study tests three different data validation methodologies with different data-partitioning strategies while developing a group method of data handling (GMDH)-based model for predicting groundwater salinity concentrations in a coastal aquifer system. The three different methods are the hold-out strategy (last and random selection), *k*-fold cross-validation, and the leave-one-out method. In addition, various combinations of data-partitioning strategies are also used while using these three validation methodologies. The prediction model’s validation results are assessed using various statistical indices such as root mean square error (RMSE), means squared error (MSE), and coefficient of determination (*R*^*2*^). The results indicate that for monitoring wells 1, 2, and 3, the hold-out (random) with 40% data partitioning strategy gave the most accurate predictive model in terms of RMSE statistical indices. Also, the results suggested that the GMDH-based models behave differently with different validation methodologies and data-partitioning strategies giving better salinity predictive capabilities. In general, the results justify that various model validation methodologies and data-partitioning strategies yield different results due to their inherent differences in how they partition the data, assess model performance, and handle sources of bias and variance. Therefore, it is important to use them in conjunction to obtain a comprehensive understanding of the groundwater salinity prediction model's behavior and performance.

## Introduction

The application of machine learning-based predictive models in the field of water resources management and engineering has increased significantly in the last decade. The availability of several machine learning algorithms, high-performing computers and infrastructures, and modeling expertise has made it easier to use machine learning-based models for water quality prediction (Ahmed et al., 2019; Liu et al., 2016), water network management (Sattar et al., 2019), water infrastructure construction (Zhang et al., 2018), and water level forecasting (Samani et al., 2023a, 2023b; Zhu et al., 2020). Developing a robust water resources prediction model is a complex task as it is dependent on the dataset used, validation methodology implied, and data-partitioning strategy applied. While the dataset for a predictive modeling task can be easily accessible, using a suitable validation and data-partitioning strategy requires attention to detail and thorough scrutiny as they both have a direct correlation to the model’s predictive capability (Kazemi et al., 2020). In this work, a first-ever comparison study is conducted where three different predictive model validation and several data-partitioning strategies are employed to develop groundwater salinity prediction models.

Predictive models are mainly used for two purposes in water resources engineering research and applications. First, predictive models are developed to predict future conditions and scenarios provided the input dataset required by the model is available (Khalil et al., 2005). Second, predictive models are used for replicating the behavior of a high‐fidelity physics‐based model or simply a complex numerical simulation model (Zahura et al., 2020). In the latter case, the predictive model is termed as the complex model’s surrogate as it can accurately mimic the complex system and provide reasonable outputs when compared to the numerical simulation model. For both purposes, predictive models need to be validated for its accuracy, efficiency, and reliability. This is one of the most challenging tasks as it requires careful consideration, modeling effort, and computational time. Model validation is the process used to decide whether the model performs satisfactorily for a problem of interest (Morrison et al., 2013). Once the model is validated for its accuracy, it can be used for its designed purpose, i.e., to either predict future conditions or accurately replicate the responses of a complex numerical simulation model. In this study, our focus is on the latter part, which is to develop and validate a predictive model capable of mimicking the responses of a complex 3D groundwater numerical simulation model.

In developing a groundwater predictive model, the standard procedure is as follows. First, the required number (user-dependent) of input and output datasets is obtained by running the complex 3D groundwater numerical simulation model. Second, this dataset is separated/divided into two sets: the model fitting set (training) and the validation set. The training set is used to fit the model, while the validation set is used to assess the performance of the trained model. Third, mathematical performance comparison indices are used to compare the performances of the outputs given by the numerical simulation model and the corresponding output from the predictive model. The biggest nontrivial question is to decide on the procedure of how to divide the data. Using appropriate standard validation methodology answers this question where multiple partitions of the data are used for model validation. The most common validation methods are the hold-out strategy (either last and random selection), *k*-fold cross-validation, and leave-one-out method, which are described next.

Hold-out strategy is one of the simplest and oldest validation methods where a portion of the selected dataset is used for fitting the model, and the leftover portion is used for validation (Pang & Jung, 2013). Often, the test set contains about 10 to 30% of the available dataset, and the fitting set contains about 90 to 70% of the dataset (Berrar, 2019). Data partitioning in the hold-out methodology can be done in two ways: (1) the last portion (certain percentage) of the dataset is used for validation, called hold-out (last); and (2) portions of the fitting and validation dataset are selected randomly (from anywhere) from the data space, called hold-out (random). In a typical *k*-fold cross-validation procedure, the dataset is randomly and evenly split into *k* parts (Valavi et al., 2018). A candidate model is built based on *k* − 1 part of the dataset, called a training set. The prediction accuracy of this candidate model is then validated using the *k* set. Using each of the *k* parts as the test set and repeating the model building and evaluation procedure, a final model is built, and its prediction capability is compared using standard comparative analysis mathematical indices. For *k* = *n*, where *n* is the total number of input–output observations in the dataset, we obtain a special case of *k*-fold cross-validation, called leave-one-out validation. In this methodology, each individual dataset, in turn, is held out for validating the model. Detailed explanations of these three validation methodologies are presented in “Predictive model validation methods.” It is critically important for predictive model developers to investigate which validation methods work best for a particular dataset. Therefore, the objective of the proposed study is to employ all three predictive model validation methodologies with different data partitioning strategies to analyze their effect on the model accuracy and computational time requirement. The interest is in presenting scientific proof that the data validation method and the number of datasets are both important for developing robust groundwater salinity prediction models.

In various hydrological studies, it is seen that a single validation methodology and a data-partitioning strategy are used for developing a predictive model. However, in some recent works, it is clearly established that it is advisable to employ different validation methodologies and data-partitioning strategies and choose the best performing model for a predictive task. For example, in a recent study, Vabalas et al. (2019) established that validation of machine learning models is imperative for developing a robust predictive model. Their study demonstrated that using one validation methodology may give rise to biased performance estimates and will not be sufficient to observe overfitting. They also suggested that meaningful comparisons of different validation methods are even more important when available training and testing samples are small. Lastly, they concluded that it is vital to utilize and compare different validations with data partitioning strategies to develop a robust predictive model regardless of the sample size. In another similar study, Morrison et al. (2013) argued that while employing one validation methodology with a single data-partitioning strategy is often used, in practice, the distinction between training and validation is not so clear and simple. Their study suggested that the available datasets must be optimally divided, and their performances compared to develop a reliable predictive model.

In addition, Wang et al. (2015) demonstrated that a predictive model cross-validation performance often relies on the quality of the data partitioning. Their study outlined that poor data partitioning may cause poor predictive results, and therefore, creating several partitions of datasets using different experimental designs and comparing their performances is a must while developing an accurate predictive model. Furthermore, Seidu et al. (2023) demonstrated different data partitioning techniques used for predicting optimum groundwater levels by different machine learning models. The authors reported that 70–30 and 80–20 data partitions gave the best groundwater level predictions.

In this work, our main aim is to apply the three most common prediction model validation methodologies and different data partitioning strategies for developing a group method of data handling (GMDH)-based predictive model and gauge their influences on the model’s prediction performances. GMDH-based prediction models have shown considerable efficiency in replicating groundwater simulation models and predicting water salinity concentrations (Lal & Datta, 2021). GMDH stands out as a superior model compared to others like artificial neural networks (ANN), long short-term memory (LSTM), and recurrent neural networks (RNN) due to its unique ability to autonomously select the optimal architecture and features for a given dataset. Unlike ANN, LSTM, and RNN, which often require manual tuning of hyperparameters and feature selection, GMDH employs a self-organizing approach that iteratively refines its structure, effectively minimizing the risk of overfitting and improving generalization performance (Ghosh & Tagore, 2017). GMDH’s ability to handle both linear and non-linear relationships within data makes it particularly versatile, outperforming ANN, LSTM, and RNN in scenarios where complex patterns and interactions exist. Additionally, GMDH exhibits greater transparency and interpretability, as its recursive structure allows for easy understanding of the underlying decision-making process, a feature often lacking in black-box models like ANN and LSTM (Sahoo & Sankaranarayanan, 2017). Overall, GMDH emerges as a powerful modeling technique that excels in automating the model selection process, providing robust performance, and offering insights into the data generation process.

A study conducted by Samani et al., (2023a, 2023b) for Chaghlondi aquifer in Iran reported that GMDH performs better in predicting qanat water flow over other machine-learning models. Amini et al. (2023) combined GMDH and Kriging to reduce the errors in groundwater salinity estimation. The authors reported that using the cross-validation approach, the GMDH models performed better than other machine learning models.

To achieve the targeted goals of the study, input and output datasets from a simulated coastal aquifer system are used to analyze the performance of the developed groundwater salinity predictive models. This study suggests that while it may be easy to choose a predictive modeling algorithm for a task, it is very challenging to choose a suitable validation methodology and an optimal data-partitioning strategy. In addition, the result of this study suggests that it is imperative to conduct different experiments using different predictive model validation and data-partitioning strategies to develop a robust predictive model. The results presented are highly significant as they validate the usefulness of employing the three different predictive model validation methodologies and the reasons behind using different data-partitioning strategies. For the first time, a study of this nature is reported in the field of water resources research. This study is the first to justify the fundamental reasons for using different validation methodologies and data-portioning strategies when developing a predictive model. The evaluation of the various methodologies and strategies for predictive model development using a coastal aquifer case study was needed to highlight how a predictive model behaves under different validation methodologies and data-partitioning strategies and why it is advisable for water resources engineers, hydrogeologists, climatologists, and water management decision-makers to not rely on a single validation methodology or data-partitioning strategy while developing predictive models.

The paper is structured as follows. The methodology including the descriptions of the various validation methodologies, data partitioning strategies, description of the GMDH algorithm, the experimental design, and the study area is presented in the “Methods and Data.” Results and discussions are presented in the “Results and discussion.” Lastly, the recommendations as well as future work and conclusions are presented in the last two sections, respectively.

## Methods and data

### 3D groundwater numerical simulation

The FEMWATER model is a three-dimensional finite element model that can simulate the flow and mass transport of both saturated and unsaturated conditions of porous media (Lin et al., 1997). For the present study, the FEMWATER package from Groundwater Modeling Systems (Aquaveo) was used to simulate pumping-induced saltwater intrusion phenomena into a coastal aquifer system. The FEMWATER modeling platform uses the Galerkin finite-element approximation and residual finite-element method to approximate flow and transport equations. Successful implementation of FEMWATER for groundwater flow and transport modeling has been reported in several studies (Koda & Wienclaw, 2005; Carneiro et al., 2010; Kim et al., 2012; Lal & Datta, 2020; Sharan et al., 2021). In developing a FEMWATER 3D model, the governing flow and transport equations can be solved depending on the specific value of a hydrogeological substance, hydraulic conductivity characteristics, initial conditions, and boundary conditions. Using the FEMWATER model, flow and transport equations are calculated simultaneously to simulate seawater intrusion. In this study, a hypothetical coastal aquifer system was simulated using the FEMWATER computer package for method evaluation and data acquisition.

### Experimental design for data acquisition

**Fig. 1:**
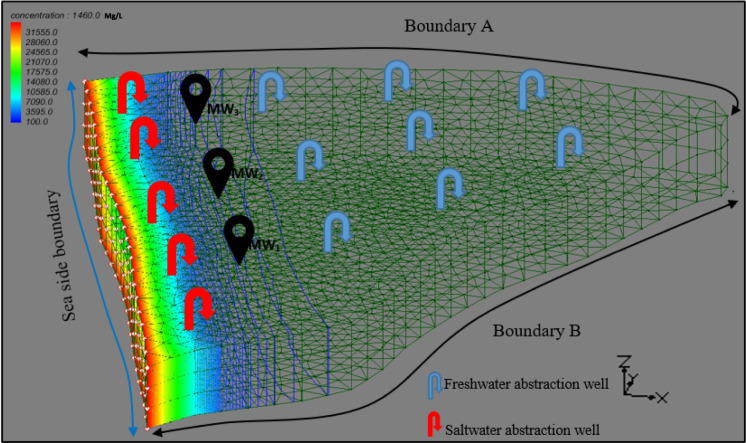
Groundwater salinity contour at the end of the 4th time step (4 years) in response to one set of pumping conditions from all groundwater and saltwater abstraction wells

The constructed 3D numerical simulation model of the study area of 2.53 km^2^ comprised a portion of a multi-layered coastal aquifer. The length of the seaside boundary, shown in Fig. 1, was 2.13 km, and the other two side boundaries were 2.04 km (*Boundary A*) and 2.79 km (*Boundary B*), respectively. The aquifer had a depth of 60 m, which was equally divided into three layers. Each layer in the aquifer had different hydrologic properties, and therefore, the aquifer was considered heterogeneous vertically. The aquifer system consisted of eight freshwater abstraction wells (FAW) and five saltwater abstraction wells (SAW) for seawater intrusion prevention located close to the seaside boundary. Saltwater abstraction from wells installed near the coastline is a common approach to controlling saltwater encroachment into fresh groundwater and has been successfully implemented in various case studies worldwide (Kallioras et al., 2012; Sharan et al., 2024; Sreekanth & Datta, 2010). Saltwater abstraction creates a trough along a shoreline, causing saltwater to flow inward and freshwater to flow in the opposite direction, i.e., toward the sea, which creates a hydraulic barrier that can reduce saltwater intrusion into freshwater systems (Sharan et al., 2023; Todd, 1974). Furthermore, Lal and Datta (2019) evaluated the benefits of SAW using a real case study aquifer system in the Pacific Islands. Their results demonstrated that the installation of SAW can serve a beneficial purpose and be regarded as a practical option for regulating saltwater intrusion. Three additional monitoring wells (MW_1_, MW_2_, and MW_3_) were installed to monitor groundwater salinity. A 3D view of the simulated aquifer system with different boundaries and well locations is given in Fig. 1. The seaside boundary had constant contact with the ocean and was assigned a constant head and constant concentration boundary (assigned concentration = 35 kg/m^3^). The other two boundaries were no-flow boundaries. The aquifer was discretized into finite triangular elements having an average element size of 150 m. The element size near the wells was reduced to 75 m, and constant groundwater recharge was specified over the entire model domain. The volumetric domain modeled by FEMWATER was idealized and discretized into a “Prism or wedge.” The elements were typically grouped into zones representing different stratigraphic units. Each element is assigned a material ID representing the zone to which the elements belong. When constructing a mesh, care was taken to ensure that elements do not cross or straddle stratigraphic boundaries. The screening interval was taken from the aquifer’s second and third layers. Various hydrologic parameters and their respective values used for the simulation are given in Table 1.

**Table 1:** Hydrologic parameters values used for 3D numerical simulation model development

Hydrologic parameters		Values used for simulation purposes
Hydraulic conductivity	*x* direction	15 m/d
	*y* direction	7.5 m/d
	*z* direction	1.5 m/d
Bulk density		1600 kg/m^3^
Longitudinal dispersivity		50 m
Lateral dispersivity		25 m
Molecular dispersion coefficient		0.69 m^2^/d
Density reference ratio		0.025
Soil porosity		0.46
Compressibility		8.5 × 10^–15^ md^2^/kg
Dynamic velocity of water		131.328 kg.md
Groundwater recharge		0.00054 m/d

The relative conductivity, moisture content, and water capacity curves are usually determined directly by performing a series of tests on the soils involved in the study. However, as done in many cases, this study approximated the curves using a set of measured or approximated constants and a set of empirical relationships. Specifically, the curves were generated using the van Genuchten functions (van Genuchten, 1980) given below.

$${K}_{r}={\theta }_{e}^{0.5}[{1-({1-{\theta }_{e}^{{~}^{1}\!\left/ \!{~}_{\gamma }\right.})}^{\gamma }]}^{2}$$

$${\theta }_{e}=[{1+({|\alpha h|)}^{\beta }]}^{-\gamma }\text{ for }h < 0$$

$${\theta }_{e}=1\text{ for h }\ge 0$$

where

$${\theta }_{w}= {\theta }_{r}+ {\theta }_{e} ({\theta }_{s}- {\theta }_{r})$$

$$\gamma =1- \frac{1}{\beta }$$

And

θ_*w*_: moisture content (dimensionless)
θ_*e*_: effective moisture content (dimensionless)
θ_*s*_: saturation moisture content (dimensionless)
θ_*r*_: residual moisture content (dimensionless)
β,γ: soil-specific exponents (dimensionless)
α: soil-specific coefficient (1/L)
*K*_*r*_: Relative Conductivity

The values for saturated and residual moisture contents and the van Genuchten α and β terms for the soil type used in the study were attained from Carsel and Parrish (1988). Also, when applying the α term, necessary care was taken to convert it to the proper units.

The 3D numerical simulation was commenced initially using a steady-state condition of the aquifer, achieved via constant pumping of 300 m^3^/day from only three of the production wells for a period of 20 years. After 20-year simulation period, it was noticed that the observed heads at different nodes in the model domain became constant. These constant heads and concentrations were used as initial conditions (initial head and concentration) to run the model for a further 4 years (*using yearly time steps*) where pumping from all FAW and SAW was instigated. This model was used to generate datasets needed for developing the GMDH-based groundwater salinity predictive models. The aquifer had 13 pumping wells (8 FAW and 5 SAW), and constant pumping from each well every year within the 4-year management time frame was instigated. This gave a total variable of 52 (13 wells × 4 years). A set of 700 randomized transient pumping (inputs) values from all FAW and SAW were obtained via Latin hypercube sampling (Loh, 1996), having an upper bound of 1300 m^3^/d and a lower bound of 0 m^3^/d. The number of input–output datasets was arbitrarily selected. For a similar illustrative coastal aquifer management problem investigated in Lal and Datta (2018), 700 pumping and concentration datasets were found to be sufficient in training and validating support vector regression surrogate models with reasonable prediction accuracy. On the other hand, in a similar saltwater intrusion modeling investigation, Yadav et al. (2017) established that only 300 input–output datasets were adequate to train an artificial neural network, support vector machine, genetic programming, and extreme machine learning models with reasonable accuracy. The number of training and testing datasets is dependent on the prediction performances of each machine learning-based predictive model type. The needed training and testing datasets can be increased or decreased depending on the prediction capabilities of the models, which can be deduced from the performance evaluation results. In the present case, 700 datasets were found to be sufficient in developing and validating the GMDH models. Each of these 700 datasets was fed to the numerical simulation model, and groundwater salinity values at respective monitoring wells were monitored. This was repeated 700 times to obtain 700 different sets of input–output patterns. Each simulation took approximately 4–5 min to converge. These input–output patterns with different validation and data partitioning strategies were later used for developing GMDH-based groundwater salinity prediction models.

### Predictive model validation methods

#### Hold-out validation (last and random selection)

**Fig. 2:**
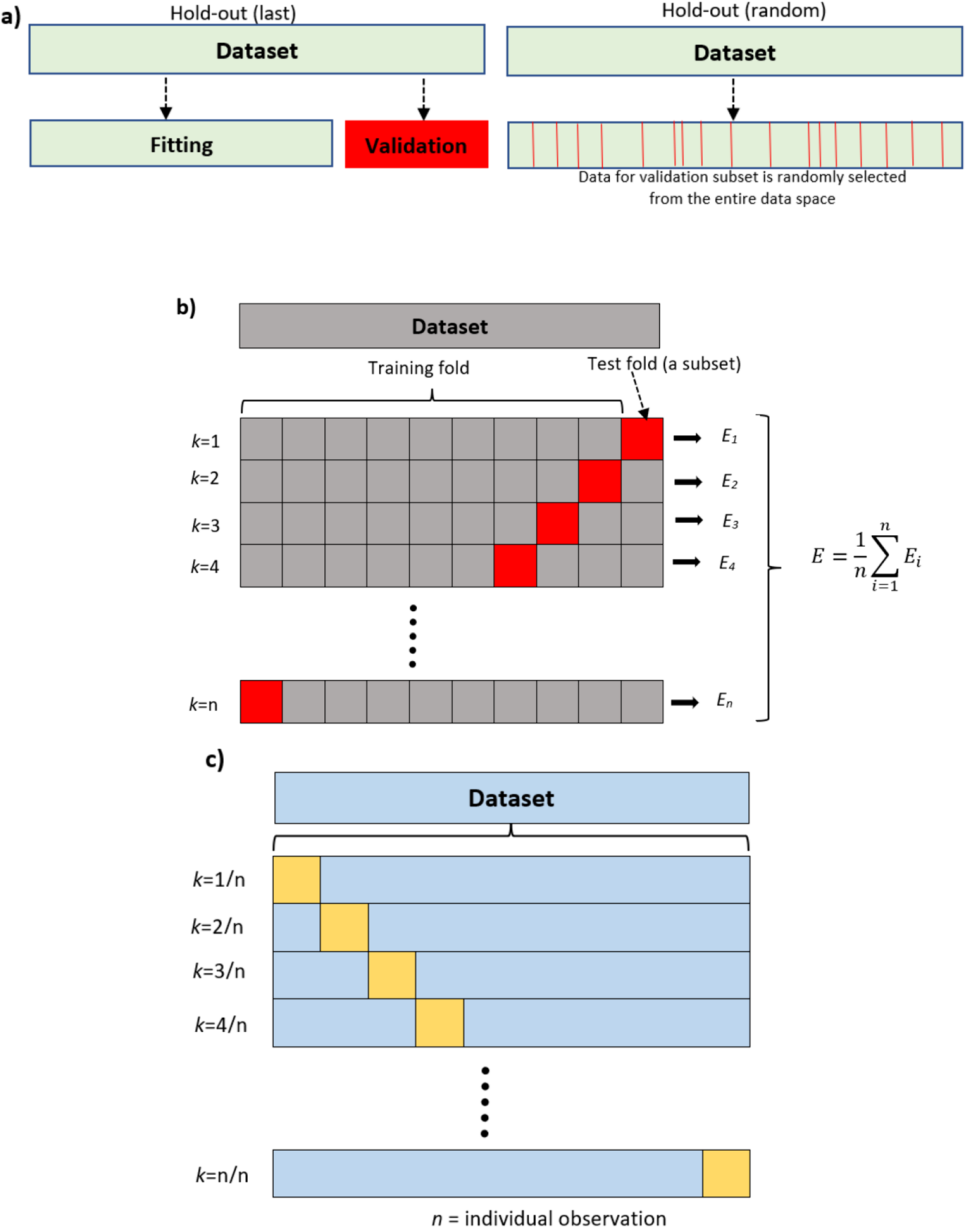
Schematic of **a** hold-out validation, **b** *k*-fold cross-validation, and **c** leave-one-out validation methodologies

The hold-out methodology is most used to validate predictive models whereby the entire dataset is divided into two different sub-sets, namely, training (model fitting) and test sets. The model is trained on the training (or fitting) sub-set data, and then it is tested using the test subset. The testing subsets allow the users to see how well the developed predictive model has performed (Molinaro et al., 2005; Kim, 2009; Kumar, 2012). The splitting/division of the entire datasets into training and testing subsets can be done in two ways. First, the last portion (usually user-specified *x%*) of the dataset can be withheld and kept separate and used as the testing dataset referred to as the hold-out (last) validation methodology. On the other hand, in some cases, the testing dataset (user-specified *x%*) can be taken randomly from the entire datasets available. This is referred to as the hold-out (random) validation methodology. Both validation methodologies are commonly used for large datasets. A simple schematic hold-out strategy is shown in Fig. 2a. Kearns (1997) has thoroughly given the overall description and a step-by-step guide on how to use the hold-out validation methodology and summarized ways in which users can minimize predictive model performance errors. In addition, Sahu and Mishra (2011) studied the performance of feed-forward neural network for novel feature selection, and their accuracy was tested using the hold-out methodology. Their results showed that support vector machine algorithm had 100% accuracy using hold-out validation.

#### k-fold cross-validation

The* k*-fold cross-validation is another commonly used validation methodology where model fitting and validation use subsets of the entire datasets. Cross-validation is typically used to improve model prediction, even though we do not have enough data points (Dantas, 2020). The validation is done in multiple layers and times whereby the entire dataset is divided into several parts, referred to as the folds. Each fold is used as the validation set at a time, while the remaining folds are used as the training set. This happens iteratively until all the folds have been used as a validation set. Mathematically, this process continues for *N* number of times, where *N* takes the value of *k*, depicting the number of validation processes conducted on the dataset. A simple *k*-fold cross-validation process is shown in Fig. 2b. Cross-validation is said to permit a high chance of detecting model over-fitting. Nurhayati et al. (2014) used hold-out and *k*-fold cross-validation for accuracy of groundwater modeling in tidal lowland reclamation using an extreme learning machine (ELM). They reported that *k*-fold cross-validation indicated a good performance of the ELM at both the training and validation stages. Numerous other studies have used the *k*-fold cross-validation methodology to evaluate the accuracy of various machine learning-based predictive models (e.g., Ahmadi et al., 2022; Borra & Ciaccio, 2010; Fushiki, 2011). These studies also reported that *k*-fold gives better accuracy than other techniques.

#### Leave-one-out validation

The model is evaluated following its name, where one observation from the entire dataset is left out for model validation. The trained model is then used to predict the response value of the one observation that was left out. A simple schematic of the leave-one-out validation methodology is presented in Fig. 2c. The leave-one-out validation methodology is slightly different from other validation methodologies as it uses the entire datasets for model validation each at a time. The leave-one-out validation methodology is renowned for its important features such as providing a less biased measure of test mean squared error compared to a single test dataset and its ability not to overestimate the test mean squared errors. Despite offering serious advantages, leave-one-out is less commonly used because there is a major drawback associated with this methodology. The leave-one-out validation methodology is established to take longer computational time, and therefore, it is computationally expensive (Zach, 2020). For example, Hawkins et al. (2003) used hold-out and leave-one-out validation methodology to check the plausibility and reliability of their QSAR model. They reported that the leave-one-out validation methodology is computationally demanding than the hold-out strategy due to the amount of time required in carrying out leave-one-out test.

These three validation methodologies with different data partitioning strategies were used for developing GMDH-based groundwater salinity predictive models. A description of the different data partitioning strategies is given in the next section.

### Data partitioning strategies

The hold-out (last), hold-out (random), and *k*-fold validation methodologies can be implemented using different subsets or folds of data, respectively. Partitioning of data into subsets and/or folds demands careful attention and consideration. Different partitioning strategies can be used for a particular modeling task, and most of the time, it is user-dependent. Different data partitioning strategies have a different impact on the predictive accuracy and the computational time requirements. In this study, for evaluation purposes, 700 datasets were divided using different partitioning strategies. The partitioning details are given in Table 2.

**Table 2:** Different data-partitioning strategies used for modeling fitting and validation

Validation methodology	Data-partitioning strategy	Data used for validation
Hold-out (last)	6 different strategies utilized	10%, 20%, 30%, 40%, 50%, and 60%
Hold-out (random)	6 different strategies utilized	10%, 20%, 30%, 40%, 50%, and 60%
*k*-fold	7 different strategies utilized	*k* = 4, *k* = 5, *k* = 6, *k* = 7, *k* = 8, *k* = 9, and *k* = 10
Leave-one-out	700 different portioning strategies utilized. Each time, 1 set is left for validation while 699 datasets are used for training	*k* = 700

### GMDH algorithm

In recent times, the GMDH algorithm has been successfully used in prediction investigations, clusterization studies, system identification, data mining, and developing knowledge extraction technologies. It was first introduced by the former Soviet scientist Ivakhnenko and is a widely used method for recognizing non-linear relationships between a set of input and output (Fernández & Lozano, 2010). In principle, the GMDH model functions by generating a high-order polynomial network, which is principally a feed-forward and multi-layer neural network. The GMDH works by providing a self-organizing data mining platform, which automatically decides the variables to be used in the modeling framework, the structure (neurons in hidden layers), and the model parameters. The model itself provides an optimal structure, which reduces the need for prior knowledge and assumptions. This feature of the GMDH algorithm reduces the possibility of any user biases and minimizes the model complexity (Xiao et al., 2017). The construction of the GMDH models requires the division of the input dataset into two groups. The first group is used to approximate the parameters of each neuron to obtain a partial description of the process, and the second group is used to weigh the performance of the candidate models that describe the process more efficiently (Fernández & Lozano, 2010). Specifically, the training dataset is used to approximate the coefficients of the Kolmogorov–Gabor polynomial, while the testing set is used in the GMDH network for error evaluation. GMDH works by constructing successive layers with connections that are the individual terms of a polynomial (Srinivasan, 2008). The output of each neuron is assessed and evaluated by an external criterion. The model disregards the neurons that has the poorest prediction results and preserves the neurons with excellent performance as the next layer. These steps are repeated to create new layers until the error criterion stops decreasing. The whole process of training and assortment is repeated on this new layer. Once neurons that best satisfy the pre-specified criterion are chosen, the model is verified using the testing dataset. A more detailed description of the GMDH modeling algorithm is available in the literature (Farlow, 1984; Liu et al., 2018; Srinivasan, 2008). Also, successful implementation of GMDH-based models for saltwater intrusion and groundwater level prediction is demonstrated in Lal and Datta (2021) and Moosavi et al., (2021), respectively. For the present assessment activity, GMDH shell software was used for model development. Different validation methodologies and data-partitioning strategies were user-dependent and manually implemented into the GMDH shell. Depending on the provided datasets, the GMDH model itself automatically decides the variables to be used in the modeling framework, the structure (neurons in hidden layers), and the models’ parameters. This is one of the significant benefits of using GMDH algorithm in predictive modeling. In addition, all the GMDH-based predictive models were developed using a single standard computer set, i.e., (Intel® Core™ i7-2600 CPU @3.40 GHz, 8 GB RAM) with RMSE as the external stopping criterion.

### Performance evaluation indices

The performance evaluation of all the GMDH models developed was evaluated during the fitting and validation phases to critically examine their efficiency in predicting groundwater salinity concentrations. Three goodness-of-fit indices (also known as “statistical indicators”) such as root mean square error (RMSE), mean absolute error (MAE), and coefficient of determination (*R*^2^) were used to evaluate the developed GMDH models. Table 3 presents a summary of these three indices.

**Table 3:** Summary of the statistical indices used for predictive model evaluation

Statistical indices	Mathematical representation	General rule
RMSE	$\text{RMSE}=\sqrt{\frac{1}{n}\sum_{i=1}^{n}{\left({c}_{T}-{c}_{P}\right)}^{2}}$	A lower RMSE value indicates a better performing predictive model
MAE	$\text{MAE}=\frac{{\sum }_{i=1}^{n}\left|{c}_{P}-{c}_{T}\right|}{n}$	A lower MAE value indicates a better performing predictive model
*R*^2^	${R}^{2}=\frac{\sum_{i=1}^{n}{(c}_{T}-\overline{{c}_{T}})({(c}_{P}-\overline{{c}_{P}})}{\sqrt{\sum_{i=1}^{n}{\left({c}_{T}-{c}_{T}\right)}^{2}} \sqrt{\sum_{i=1}^{n}{\left({c}_{P}-{c}_{P}\right)}^{2}}}$	A higher *R*^2^ value indicates a better performing predictive model

Where $n$ represents the total number of datasets, ${c}_{T}$ is the true concentration from the numerical simulation model, ${c}_{P}$ represents GMDH predicted concentrations, $\overline{{c}_{T}}$ is the mean true concentration from the numerical simulation model, and $\overline{{c}_{P}}$ denotes mean GMDH predicted concentrations

## Results and discussion

### Hold-out (last vs. random) validation comparison

**Fig. 3:**
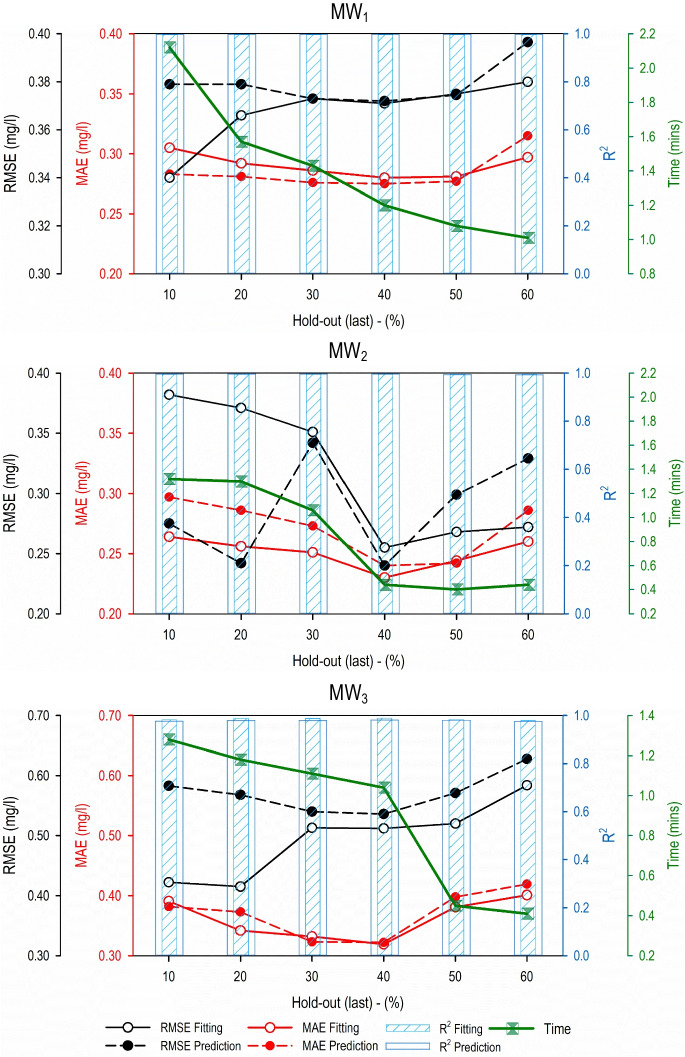
Performance evaluation results for the predictive models developed using the hold-out (last) validation methodology and different data partitioning strategies

**Fig. 4:**
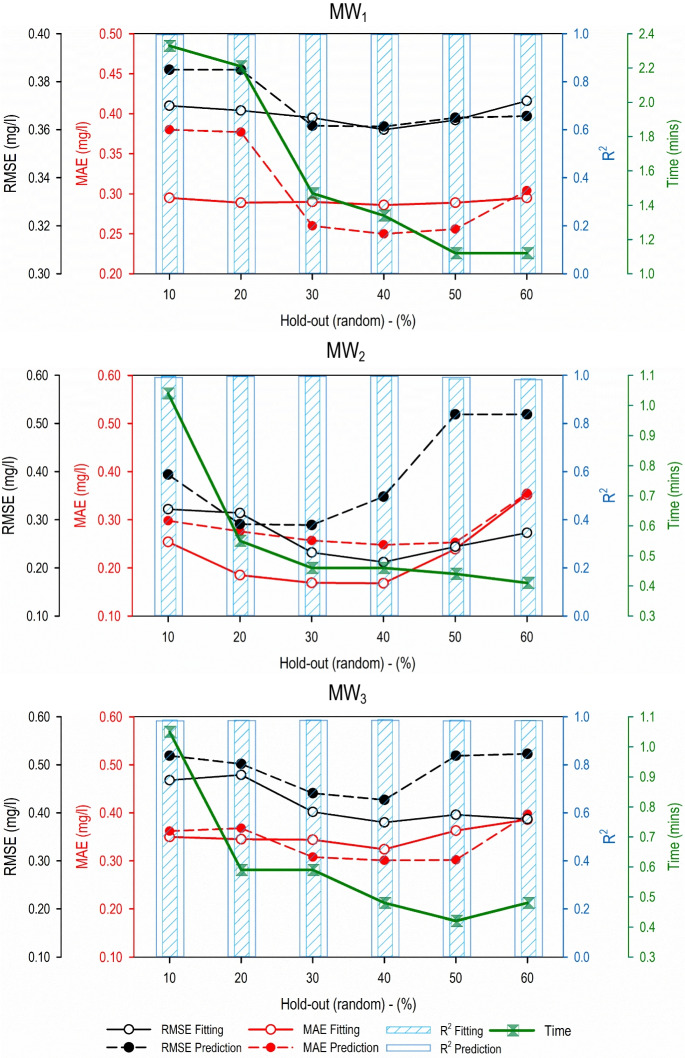
Performance evaluation results for the predictive models developed using the hold-out (random) validation methodology and different data partitioning strategies

The results for the hold-out (last) and hold-out (random) validation methodologies in terms of RMSE, MAE, *R*^2^, and computational time are presented in Figs. 3 and 4, respectively.

For MW_1_, it is observed that for both the fitting and validation phases, hold-out (last)–40% gave the best predictive accuracy results in terms of RMSE and MAE indices. The lowest value of RMSE and MAE is obtained during this validation and data-partitioning strategy. The *R*^2^ value remained the same for all six data-partitioning strategies. The computational time requirement decreased as the subset of the validation dataset increased, i.e., as the hold-out (last)–40% increased. Similar results are recorded for MW_2_ and MW_3_, i.e., hold-out (last)–40% gave the most accurate GMDH-based models in terms of RMSE and MAE. Also, similar to MW_1_, the computational time requirement for model fitting and validation decreased as the validation subset increased.

For the hold-out (random) validation methodology, it is observed that for both the fitting and validation phases, the data partitioning strategy of 40% gave the best accuracy results in terms of RMSE and MAE for all three monitoring wells. The lowest values of RMSE and MAE were recorded for models developed using the hold-out (random)–40% validation and data-partitioning strategy. The *R*^2^ values showed no particular trend as they fluctuated between 0.996 to 0.997, 0.982 to 0.996, and 0.983 to 0.985 for MW_1_, MW_2_, and MW_3_, respectively. In addition, similar to hold-out (last) validation methodology, the computational time requirement decreased as the subset of validation dataset increased, i.e., when the hold-out (random) dataset for validation increased from 10 to 60%. This was true for all three monitoring wells.

In general, for the present case study, both hold-out (last)–40% and hold-out (random)–40% gave the best performing predictive model and can be used for groundwater salinity prediction at the three monitoring wells. On the other hand, if computation time is considered important over the accuracy, then hold-out (last)–60% and hold-out (random)–60% data-partitioning strategy is to be used.

### *k*-fold cross-validation comparison

**Fig. 5:**
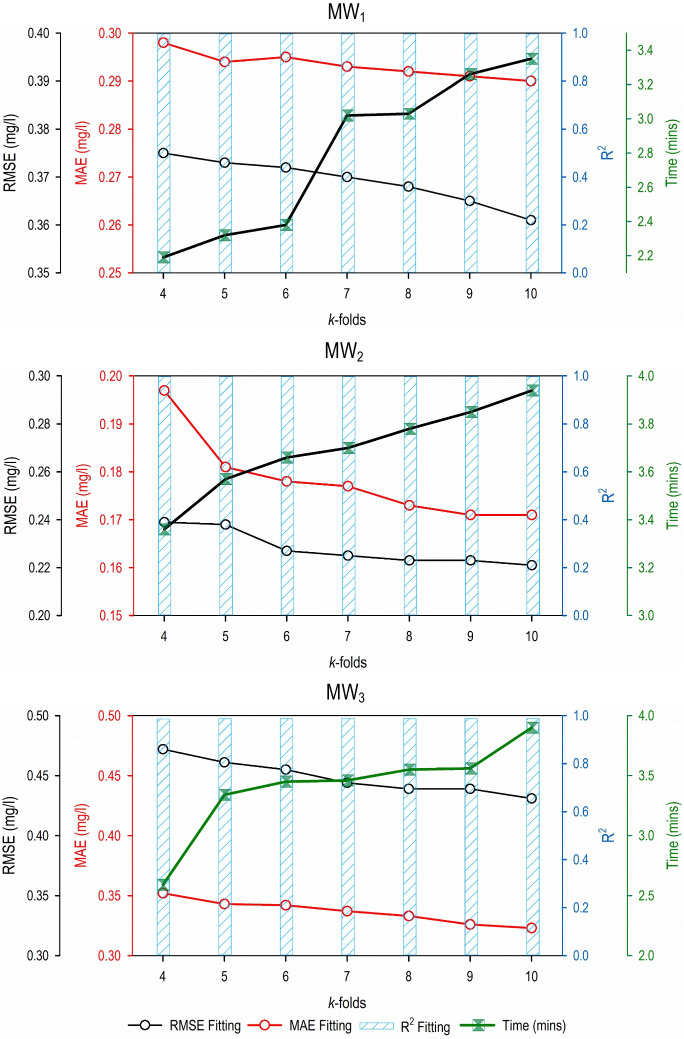
Performance evaluation results for the three predictive models developed using the* k*-fold validation methodology and different data partitioning strategies during the fitting phase

The *k*-fold cross-validation results are presented in Fig. 5. All the developed models show a similar trend in terms of accuracy and computational time requirements. For example, RMSE and MAE values declined as the fold increased from *k* = 4 to *k* = 10. This was true for all three monitoring wells. Also, it was observed that the computational time requirement increased as the folds increased from *k* = 4 to *k* = 10. The values for* R*^2^ did not show such trend as the values remained the same. Overall, these results establish that a predictive model’s accuracy increases as the number of folds increases. Therefore, a larger value of* k* should be used when deciding to use *k*-fold validation for a predictive modeling task. However, it is also important to consider the computational time as another factor given it increases with more folds (i.e., more *k*). There is always a trade-off between accuracy and computational time requirement, and an optimal number of *k* is always dependent on the user. Therefore, several trials need to be conducted with different values of *k* before selecting a particular model for a modeling purpose.

### Leave-one-out comparison

**Fig. 6:**
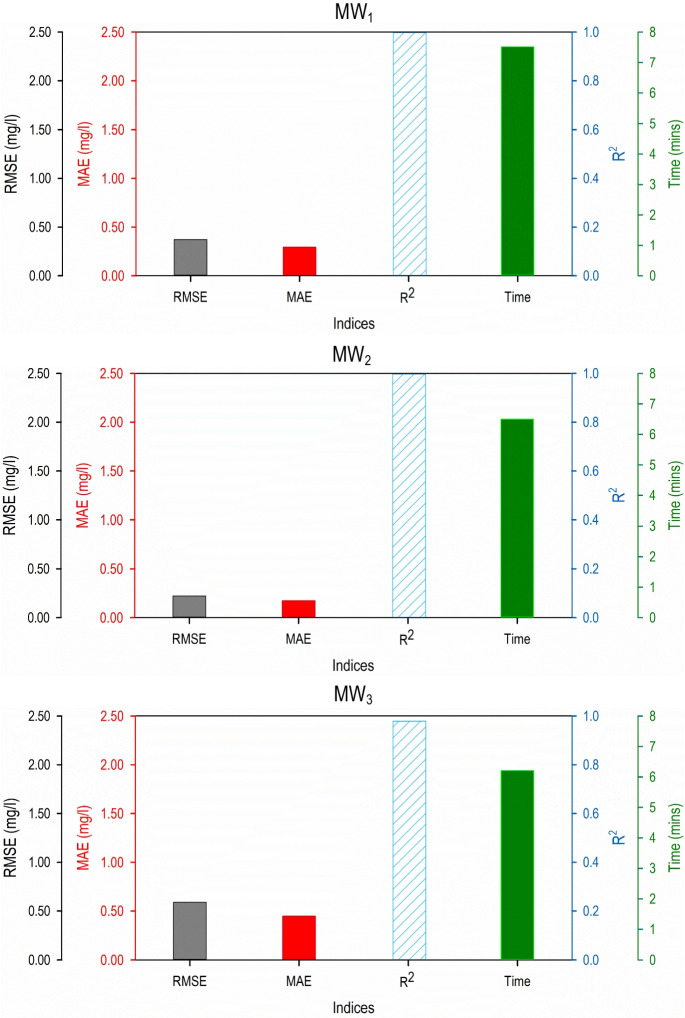
Performance evaluation results for the 3 predictive models developed using the leave-one-out validation methodology during the fitting phase

The performance evaluation results in terms of the different statistical indices for the GMDH-based predictive models, fitted, and validated using the leave-one-out validation methodology are presented in Fig. 6.

As per Fig. 6, the predictive model developed using the leave-one-out methodology had comparable results in terms of accuracy when compared to the other two validation methodologies. In most cases, the RMSE and MAE values obtained for the predictive models during leave-one-out validation were higher than the respective values obtained for hold-out and *k*-fold validation methodology. However, there were also instances when leave-one-out method prediction results were better than other validation methods. For, e.g., the fitting RMSE values for leave-one-out and *k*-fold (*k* = 4) were 0.372 mg/l and 0.385 mg/l, respectively. A similar trend was seen in MAE values. These results demonstrated that leave-one-out method can perform better in certain cases and should be used for comparison. The results obtained also justify the fact that different validation and data-partitioning strategies have different inferences of the accuracy of a predictive model. The *R*^2^ values have minimal variations when compared to the other two validation methodologies. This is true for the results for all three monitoring locations. On the other hand, the computational time taken by leave-one-out is significantly higher than the other two validation methodologies. The time taken for developing GMDH-based predictive models for MW_1_, MW_2_, and MW_3_ are 7.52 min, 6.50 min, and 6.21 min, respectively. These values are much higher than the corresponding computational time obtained using the other two validation methodologies. This highlights that the leave-one-out methodology is time-consuming and may not be preferred over the other two validation methodologies.

### Selection of the best possible predictive model—trade-off investigation

**Fig. 7:**
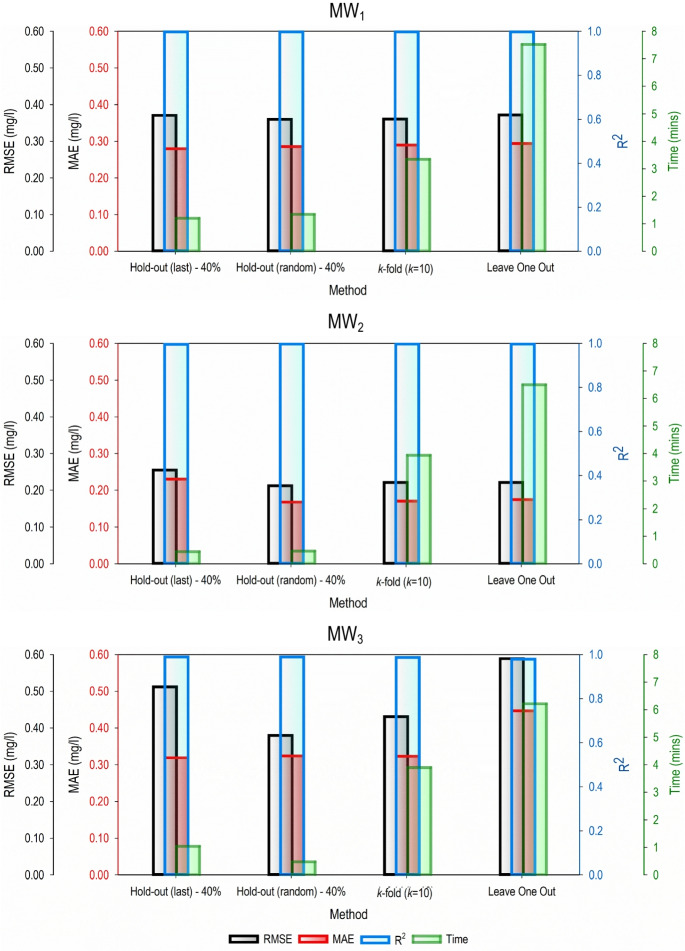
Performance evaluation results of the best performing models

Selecting the best model for the predictive task is quite challenging. It involves analyzing different trade-offs between accuracy and computational time. It depends on the need of the modeling investigation and/or the user. Sometimes, higher accuracy is preferred over computational time. This is when the reliability of the predictive model in providing accurate or precise modeling results is of utmost importance. However, computational time is given more weight than accuracy in some cases, for instance, in real-time systems in vehicles and industrial control systems. In the present case, it is observed that no validation methodology and data partitioning strategy give similar predictive accuracy results. Also, the computational time for each of the methodologies is different. For evaluation purposes, the best performing models in each of the three validation methodologies are presented in Fig. 7. The methods and machine learning models utilized in this study could be used for other aquifers. However, the numerical model needs to be developed with the corresponding aquifers hydrogeological parameters.

Figure 7 demonstrates that it is difficult to choose a particular predictive model for groundwater salinity prediction for the simulated aquifer system. All four validation methodologies possess different accuracy and require different computational time.

For MW_1_, in terms of RMSE, it is observed that hold-out (random)–40% data partitioning strategy gave the most accurate predictive model. However, the best predictive model was obtained by hold-out (last)–40% in terms of MAE. The values of *R*^2^ obtained for all the methodologies were the same. Lastly, the computational time required for each model was different, with the highest time obtained for leave-one-out and lowest time obtained for hold-out (last)–40% data partitioning strategy. For MW_2_, the best predictive model was obtained using hold-out (random)–40% as demonstrated by their RMSE and MSE, whereas hold-out (random)–40% had the lowest value for the same indices. *R*^2^ values did not show much of a difference. The prediction model developed using hold-out (last)–40% had the lowest computational time requirement of 0.44 min, while the leave-one-out validation methodology took the longest computational time of 6.5 min. Similarly, for MW_3_, hold-out (random)–40% had the lowest RMSE value of 0.38 mg/l, whereas the lowest MAE value of 0.319 mg/l was obtained for hold-out (last)–40%. Hold-out (random)–40% and hold-out (last)–40% also gave the best results in terms of *R*^2^. Lastly, hold-out (random)–40% model took the shortest time, while the leave-one-out methodology took the longest computational time. Overall, no particular trend is deduced as it is observed that different validation methodologies and data partitioning strategies behaved differently when used on the same 700 input–output dataset. However, it can be established that both in terms of RMSE, MAE, and computational time requirements, the hold-out validation methodology with 40% data partitioning strategy produced better prediction result.

## Recommendations and future work

After analyzing the results of this study, the following recommendations can be made.

1. Different validation methodologies employed for predictive model fitting and validation perform differently. Users should validate the predictive accuracy of models using each of the available validation methodology.
2. Dataset partitioning strategies used for model validation also influence the accuracy of predictive models. It is important to have a comparative assessment of different data partitioning strategies before agreeing to use a single strategy. This is particularly critical when developing improved and robust prediction models.
3. The computational time requirement for model fitting and validation also differs for different validation methodologies and data partitioning strategies.
4. There is always a trade-off between accuracy and computational time. The selection of a predictive model validation methodology and data partitioning strategy is dependent on user preference and predictive modeling aim.
5. Different numerical codes would be used to develop numerical models, for instance, MODFLOW, MT3DMS, and SEAWAT. Then comparing the results with FEMWATER would be more debatable.
6. Carrying sensitivity analysis while changing model input parameters would be considered in the future.

The main goal of this study was to demonstrate the effectiveness of the three most common prediction model validation methodologies and different data partitioning strategies on the predictive callability of a prediction model. While this study has demonstrated several novel results and outcomes, there are a few limitations. One limitation of the study is that the utilized GMDH-based predictive models is a black-box model, which fails to simulate the internal physical processes of saltwater intrusion. The predictive model only learns to approximate the system and is dependent on the input–output dataset used in its development. Therefore, the GMDH-based predictive models should not be used for understanding the underlying saltwater intrusion processes in the investigated aquifer system. A robust 3D numerical simulation model should be utilized for this purpose. The second limitation of the present work is that it only uses a single machine learning algorithm, i.e., GMDH for replicating the aquifer system and predicting salt concentrations at respective monitoring locations. Other state-of-the-art modeling algorithms and machine learning models could be used, which might yield different prediction results. However, this was not in the scope of the present study. In the near future, the authors have planned to verify the methodology using different machine learning algorithms such as polynomial chaos expansion (PCE) and multivariate adaptive regression splines (MARS). It would be interesting to compare the performance evaluation results of the developed GMDH models with these models. It is anticipated that different modeling algorithms might perform differently on the same dataset and yield different results in terms of accuracy and computational time. In addition, the authors have also planned for a similar study where the performances of predictive models developed using less than 700 datasets will be assessed and compared. If the results are similar or comparable, then fewer datasets can be used for similar modeling investigations saving us computational time and effort. Third, the utilized GMDH model may not be able to accurately replicate the system and predict salt concentration when the number of dimensions in the problem variable space is large. In the present work, GMDH performs reasonably well with 52 variables. However, this may change when the number of variables increases, i.e., when more FAW and SAW are considered. In this case, a different predictive modeling algorithm capable of handling large variable size could be utilized. Lastly, other sophisticated statistical indices can be used to assess the accuracy of the developed predictive models. These additional indices can help us verify and confirm the accuracy indicated by RMSE, MAE, and *R*^2^ values.

## Conclusions

Accurate fitting and validation are indeed one of the most important stages during the development of a predictive model. This study used three different validation methods and several data-partitioning strategies to develop a robust GMDH-based groundwater salinity prediction model. The analysis carried out in the study established that GMDH-based model’s predictive performances were comparable to the 3D numerical simulation model. However, as illustrated in this case study, careful understanding of the validation processes and as well as an assessment of the various data partitioning strategies are required while developing accurate predictive models. The results presented in this paper are also particularly useful for real-world applications as it highlights the importance of assessing different validation methodologies and data partitioning strategies during the predictive model development stage. Noteworthy, the new insights presented in this paper are significant for hydrologists, water engineers, and other impact communities that need robust and reliable predictive models. Also, the key findings presented in this study can provide a reference for further experimental work in machine learning-based hydrological investigations.

## Data Availability

Datasets and computer codes will be made available upon request.
